# Modified Weekly Cisplatin-Based Chemotherapy Is Acceptable in Postoperative Concurrent Chemoradiotherapy for Locally Advanced Head and Neck Cancer

**DOI:** 10.1155/2015/307576

**Published:** 2015-02-22

**Authors:** Hsueh-Ju Lu, Chao-Chun Yang, Ling-Wei Wang, Pen-Yuan Chu, Shyh-Kuan Tai, Ming-Huang Chen, Muh-Hwa Yang, Peter Mu-Hsin Chang

**Affiliations:** ^1^Division of Hematology and Oncology, Show Chwan Memorial Hospital, Changhua, Taiwan; ^2^Faculty of Medicine, National Yang Ming University, Taipei, Taiwan; ^3^Program in Molecular Medicine, School of Life Sciences, National Yang-Ming University, Taipei, Taiwan; ^4^Division of Hematology and Oncology, Department of Medicine, Taipei Veterans General Hospital, No. 201, Section 2, Shih-Pai Road, Taipei 112, Taiwan; ^5^Department of Medicine,Taipei City Hospital,Yang-Ming Branch, Taipei, Taiwan; ^6^Department of Oncology Medicine, Taipei Veterans General Hospital, Taipei, Taiwan; ^7^Department of Otolaryngology, Taipei Veterans General Hospital, Taipei, Taiwan; ^8^Institute of Clinical Medicine, National Yang Ming University, Taipei, Taiwan

## Abstract

*Background*. Triweekly cisplatin-based postoperative concurrent chemoradiotherapy (CCRT) has high intolerance and toxicities in locally advanced head and neck cancer (LAHNC). We evaluated the effect of a modified weekly cisplatin-based chemotherapy in postoperative CCRT. *Methods*. A total of 117 patients with LAHNC were enrolled between December 2007 and December 2012. Survival, compliance/adverse events, and independent prognostic factors were analyzed. *Results*. Median follow-up time was 30.0 (3.1–73.0) months. Most patients completed the entire course of postoperative CCRT (radiotherapy ≥ 60 Gy, 94.9%; ≥6 times weekly chemotherapy, 75.2%). Only 17.1% patients required hospital admission. The most common adverse effect was grade 3/4 mucositis (28.2%). No patient died due to protocol-related adverse effects. Multivariate analysis revealed the following independent prognostic factors: oropharyngeal cancer, extracapsular spread, and total radiation dose. Two-year progression-free survival and overall survival rates were 70.9% and 79.5%, respectively. *Conclusion*. Modified weekly cisplatin-based chemotherapy is an acceptable regimen in postoperative CCRT for LAHNC.

## 1. Introduction

Locoregional recurrence and distant metastases are frequent after surgical resection for locally advanced head and neck cancer (LAHNC), especially for patients with poor prognostic factors, such as involvement of two or more regional lymph nodes, extracapsular spread of disease, or microscopically involved mucosal margins of resection [1, 2]. Previous studies have demonstrated that the potential value of postoperative concurrent chemoradiotherapy (CCRT) for patients with high-risk operable HNC is strong, and the results have shown benefit on locoregional control and disease-free survival [3]. However, because this postoperative CCRT regimen has the disadvantages of intolerance and poor compliance, the applicability of a modified regimen should be reevaluated.

In previous studies, triweekly high-dose cisplatin (100 mg/m^2^ per 3 weeks for three cycles) was recommended for use in postoperative CCRT [1, 2]. Although this regimen significantly improved locoregional control and survival, the compliance of these patients was very low. Only 50–60% of patients were able to receive the complete three cycles of high-dose cisplatin [1, 2], because the intensity of this regimen was poorly* tolerated* with significantly increased severe adverse effects [1]. Therefore, despite the demonstrated benefit of high-dose cisplatin [1, 2], its use is of concern to many clinicians [4]. There have been only a few studies showing that a weekly cisplatin-based regimen could be an acceptable and promising standard treatment in the definitive CCRT setting, and these studies have shown that this treatment has good efficacy and compliance with less toxicity [5–9]. And rare studies described the efficacy and adverse effects of modified weekly chemoregimen in postoperative CCRT. In Bachaud et al. article, it was a prospective randomized trial and compared the efficacy of postoperative radiotherapy with or without weekly cisplatin [10]. The result showed the combination of radiotherapy and chemotherapy in postoperative treatment for high-risk LAHNC had better disease-free survival (DFS) and overall survival (OS) than those with radiotherapy alone (2-year DFS: 68% versus 44%, *P* < 0.02; 2-year OS: 72% versus 46%, *P* < 0.01) [10], but the optimal chemotherapy regimen in postoperative CCRT remains unknown [11, 12].

To decrease adverse effects and increase compliance in postoperative CCRT, a modified chemotherapy regimen, such as the weekly cisplatin-based regimen, might have a similar efficacy, less toxicity, and better compliance [4]. Although clinical trials of modified chemotherapy regimens in the definitive CCRT setting have not had a control group treated with conventional triweekly regimen, our experience is that weekly cisplatin-based chemotherapy has not been inferior to conventional therapy and that the toxicity has been acceptable [7, 8]. Moreover, it seems that a modified weekly cisplatin-based regimen is acceptable in the postoperative CCRT setting.

We added the oral agent tegafur-uracil (UFUR; TTY Biopharm, Taipei, Taiwan) as the radiosensitizer. The pharmacokinetics of tegafur-uracil show that this drug combination is not inferior to continuous 5-FU infusion [13]. Because uracil inhibits dihydropyrimidine dehydrogenase (DPD), the concentration of 5-FU from the absorbed 5-FU prodrug tegafur increases* in vivo*, enhancing antitumor activity [14]. In addition, the use of oral tegafur-uracil avoids the complications of continuous 5-FU infusion and enables the patient to undergo radiosensitizing chemotherapy at home. Several articles have shown that CCRT regimens with tegafur-uracil are effective [6–8].

In this study, we analyzed patients diagnosed with high-risk LAHNC at Taipei Veterans General Hospital between December 2007 and December 2012. Only patients who received a modified chemotherapy regimen of weekly cisplatin/tegafur-uracil (UFUR) in postoperative CCRT were enrolled in our study. We analyzed the survival, compliance, and adverse effects of these patients. The results of this study could lead to more choice in the chemotherapy regimen for postoperative CCRT.

## 2. Materials and Methods

### 2.1. Study Design, Setting, and Patient Selection

The study was a single-institution, retrospective, cohort study. It was reviewed and approved by the Institutional Review Board of Taipei Veterans General Hospital (number 2014-05-002BC). Between December 1, 2007, and December 31, 2012, patients diagnosed with high-risk squamous cell carcinoma (SqCC) of LAHNC at Taipei Veterans General Hospital were selected. All patients underwent complete tumor resection. High-risk LAHNC was defined as having at least one major risk factor or two minor risk factors. Major risk factors included positive resection margin, extracapsular nodal spread, and the formation of tumor emboli. Minor risk factors included upstaging to a pT4 primary, pN2/N3 nodal disease, nodal disease in levels IV/V, perineural invasion, and lymphovascular invasion.

Basic clinicopathologic parameters were recorded, including age, sex, pathologic stage, primary site of tumor, and pathologic features of the tumor (e.g., differentiation of tumor, extracapsular nodal spread, status of resection margin, formation of tumor emboli, regional lymph node involvement, perineural invasion, and lymphovascular invasion).

### 2.2. Radiotherapy

All patients received postoperative curative radiation to the primary tumor at a dose of 60–66 Gy, administered as 1.8–2 Gy per day 5 days per week. The dose administered to uninvolved lymph nodes was between 44 and 60 Gy. Involved lymph nodes received 60–66 Gy [1, 2]. In general, radiotherapy was performed using the intensity-modulated radiotherapy technique. Treatment planning was performed using the Eclipse system, version 6 (Varian Medical Systems, Inc., Palo Alto, CA, USA). The gross tumor volume (GTV) was defined as any visible tumor on imaging studies and/or physical examination. The high-risk clinical tumor volume (CTV_H) encompassed the GTV with a 5–10 mm margin, including the nodal regions in the neck at levels I–IV. The low-risk CTV (CTV_L) included the clinically uninvolved contralateral neck and base of the skull. The retropharyngeal region was also included as part of the CTV in patients who presented with clinically involved neck nodes as well as in those who had primary oropharyngeal or hypopharyngeal tumors. An intermediate risk CTV (CTV_M) was determined by the treating physician for areas with a risk that was between that of CTV_L and that of CTV_H. The planning target volumes (PTV_H, M, and L) encompassed the corresponding CTVs plus a 3 mm margin. The PTV was modified if indicated (e.g., in cases where it was close to critical organs) [15].

### 2.3. Chemotherapy

During radiation, chemotherapy was administered as follows: cisplatin (25 mg per square meter of body-surface area) was infused for 2 hours on day 1, and oral tegafur-uracil (UFUR) (2 capsules twice per day) was given on days 1–7. This cycle of chemotherapy was repeated every week until the completion of radiotherapy.

### 2.4. p16^INK4A^ Immunohistochemistry

p16^INK4A^ is a well-known tumor suppressor protein encoded by three exons of the p16 gene. This gene is a member of the INK4 class of cell-cycle inhibitors. It regulates the Rb tumor suppressor pathway by keeping Rb in a hypophosphorylated state, which further promotes the binding of E2F to achieve G1 cell-cycle arrest. So, immunohistochemistry of p16^INK4A^ has been recently proposed as a screening method for HPV protein elaboration to detect a biologically distinct entity of HPV-related HNC which had been shown to have a better prognosis [16, 17]. A slide with a representative tumor was selected, and a 4 mm × 4 mm section of the slide was indicated by a board-certified pathologist. p16^INK4A^ immunohistochemistry was performed using the avidin-biotin complex technique. The degree of nuclear staining was analyzed to assess 16^INK4A^ expression. The immunostaining was graded and scored as follows: (1) <5% of the cells were positive; (2) 5–20% were positive; (3) 21–50% were positive; and (4) >50% were positive. Only a score of 4 was considered positive for p16^INK4A^ [16].

### 2.5. Follow-Up

Progression-free survival (PFS) was calculated from the date of disease diagnosis to the date of any type of progression (local, regional, metastatic, or secondary primary) or death from any cause. Overall survival (OS) was calculated from the date of disease diagnosis to the date of death or the date on which the patient was last evaluated. Compliance and treatment-related adverse effects were retrospectively recorded the events during the total course of postoperative CCRT according to the National Cancer Institute's Common Terminology Criteria for Adverse Events version 3.0 (CTCAE v 3.0) [18]. The final follow-up date was March 31, 2014.

### 2.6. Statistical Analysis

The correlations among variables were expressed as a number (percent) for categorical variables. The Cox proportional hazards model was applied for univariate and multivariate analyses. Survival was estimated using the Kaplan-Meier method. Variables with *P* values <0.05 in univariate analyses were entered into multivariate analysis models. A two-sided *P* value <0.05 was regarded as statistically significant. SPSS statistical software (version 18.0, SPSS Inc., Chicago, IL, USA) was used for all statistical analyses.

## 3. Results

### 3.1. Patient Characteristics

Between December 1, 2007, and December 31, 2012, 117 patients with high-risk HNC were diagnosed in our institution. The majority of patients (86.3%, 101/117) were younger than 65 years old (86.3%, 101/117) and predominantly male (95.7%, 112/117). Patient characteristics are shown in Table 1. Median follow-up time was 30.0 (3.1–73.0) months.

### 3.2. Compliance and Treatment-Related Adverse Effects

Most patients were able to receive radiotherapy ≥60 Gy (94.9%, 111/117) and weekly chemotherapy for six or more cycles (75.2%, 88/117). Only 17.1% patients (20/117) required hospital admission during the course of postoperative CCRT (Table 2). During the course of postoperative CCRT, we would reduce 10% dose intensity of chemotherapy if severe complication or intolerance. Only 9.4% (11/117) of patients reduce dose due to severe mucositis, fatigue, or neutropenia. After reducing 10% dose intensity, almost these 11 patients (90.1%, 10/11) were still able to receive weekly chemotherapy for six or more cycles.

The incidences of treatment-related adverse effects are shown in Table 3. Grade 3/4 mucositis was the most common adverse effect (28.2%, 33/117). Other adverse effects, such as febrile neutropenia, thrombocytopenia, nausea/vomiting, skin lesions, and anorexia, were rare and manageable. Rare incidence of xerostomia was found during the acute phase of CCRT. No patient died due to protocol-related adverse effects. Only one patient died within 30 days after the end of treatment because of severe pneumonia.

### 3.3. Univariate and Multivariate Cox Regression Analysis for the Prognostic Factors of Overall Survival

Univariate analyses revealed that location of tumor in the oropharynx (*P* = 0.014), extracapsular spread (*P* = 0.012), and total radiation dose ≥60 Gy (*P* = 0.012) were important prognostic factors. Further multivariate analyses indicated that location of tumor in the oropharynx (hazard ratio (HR), 0.261; 95% confidence interval (CI), 0.116–0.586; *P* < 0.001), extracapsular spread (HR, 2.709; 95% CI, 1.312–5.592; *P* = 0.007), and total radiation dose (HR, 0.241; 95% CI, 0.086–0.679; *P* = 0.007) were independent prognostic factors (Table 4).

### 3.4. Survival and Comparisons to Previous Studies

**Figure 1:**
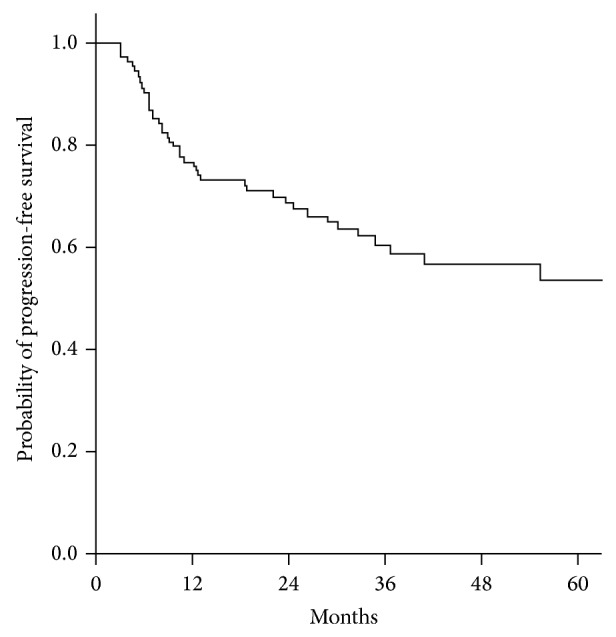
Kaplan-Meier plot of progression-free survival. The two-year progression-free survival rate of patients treated with weekly cisplatin-based chemotherapy in postoperative CCRT is 70.9%.

**Figure 2:**
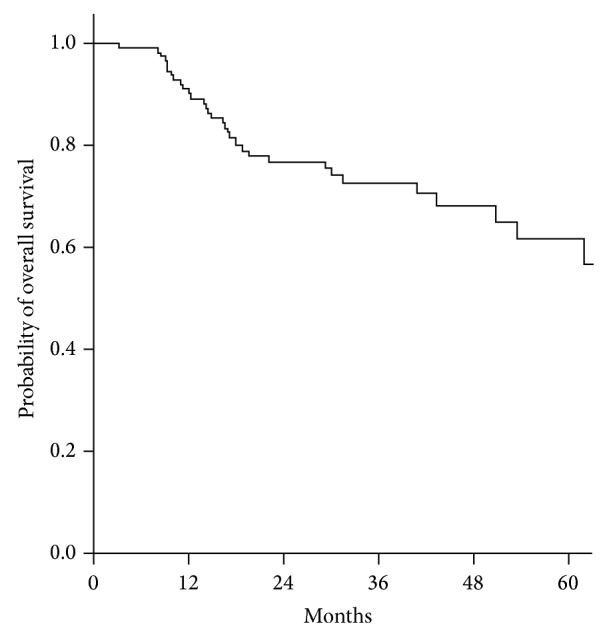
Kaplan-Meier plot of overall survival. Two-year overall survival rate is 79.5%.

With the weekly chemotherapy regimen of our study, 2-year PFS and OS rates were 70.9% and 79.5%, respectively. The Kaplan-Meier plots of PFS and OS are shown in Figures 1 and 2. Although there was no control group, the results of survival do not seem inferior to those of previous studies (Table 5).

## 4. Discussion

In our study, we analyzed the outcomes of a weekly cisplatin-based regimen in the postoperative CCRT setting. The results showed that the* efficacy* of this regimen was not inferior to the standard regimens with high-dose cisplatin and that the compliance and adverse effects were significantly improved with this treatment. The independent prognostic factors identified were the location of the primary tumor in the oropharynx, extracapsular spread, and total radiation dose. Without a randomized comparison between the standard high-dose regimen and the modified weekly regimen, however, this study was only able to demonstrate that a modified weekly regimen is feasible and effectively improved compliance/adverse effects.

The outpatient management of patients with HNC is important, because of potential changes in appearance following surgery, self-image, occupational status, and perception of social relationships and because coping skills may be altered [19], which can result in emotional distress [20]. Adequate social support has been shown to improve this emotional distress [19, 21]. Outpatient services might provide social support such as companionship [19], which would effectively improve a patient's emotional distress and quality of life [22–24]. The comparison between inpatients and outpatients with HNC has also shown that outpatient chemotherapy is reliable and cost effective [25]. In our study, the modified regimen was shown to maintain good treatment efficacy while effectively decreasing the duration of hospital admission.

Many studies have shown that the use of multiagents with 5-FU infusion-based CCRT improves the radiosensitization of tumor cells and can increase systemic activity [9, 26], but infusion of 5-FU is associated with an increased frequency of toxic effects [27]. To decrease adverse events without compromising antitumor activity, many studies have shown that tegafur-uracil- (UFUR-) based regimens in CCRT are feasible [6–8, 28, 29]. In our institute, a weekly cisplatin-based chemotherapy regimen in definitive CCRT has been shown to be acceptable and safe for treating patients with LAHNC [7, 8]. Grade 3/4 adverse effects, including neutropenia (18%, 6/33), oral mucositis (18%, 6/33), dysphagia/esophagitis (15%, 5/33), and anorexia (24%, 8/33), were manageable [7]. After comparison to our previous study [7], the current study seems to have less adverse effects. It might be due to the difference of study design and dose intensity between these two studies. In the previous phase II study [7], the chemoregimen of weekly cisplatin and tegafur-uracil (UFUR) were used as definitive CCRT setting. Patients received weekly cisplatin 30 mg/m^2^ infusion for 2 hours on day 1 and oral tegafur-uracil (UFUR) 250 mg/m^2^/day on days 1–5 repeated every week, combined with radiotherapy 70 Gy for primary tumor for a total of 7 weeks as definitive CCRT. But, in the current study, patients were treated with relatively less dose of radiotherapy (less than 66 Gy) and chemotherapy (weekly cisplatin 25 mg/m^2^) than the previous study. On the other hand, there is inevitable missing data in the record of toxicities for such retrospective analysis. Although there have been no large randomized controlled trials that have compared the* efficacy* and adverse effects between weekly cisplatin combined with tegafur-uracil and conventional triweekly cisplatin as chemotherapy for CCRT, our study was useful in that it demonstrated that weekly cisplatin-based chemotherapy could be a manageable chemotherapy regimen.

There were some limitations to this study. The first was insufficient follow-up time. In previous studies [1, 2], follow-up took place for at least five years. Although survival rate, compliance, and adverse effects in our study were better than those outcomes with conventional treatment [1, 2], a longer follow-up time is required to show long-term survival. A second limitation was that there was no comparison group in this study. Further studies using comparison groups are needed to confirm the efficiency and long-term outcomes of this modified chemotherapy regimen. Third, for patients diagnosed with locally advanced oropharyngeal carcinoma, organ preservation plays a critical role of treatment goal in our institution. Most of these patients received definitive CCRT but not radical resection followed by postoperative CCRT as the curative management, so there may be inadequate tissue for p16^INK4A^ stain. However, among 18 patients who were tested p16^INK4A^ stain, there were six hypopharynx patients, eleven oral cavity patients, and one oropharynx patient. So, it needs more experience in the future to discuss the issue of HPV-related LAHNC.

However, modified weekly cisplatin-based chemotherapy should be considered in clinical practice.

## 5. Conclusion

In our study, the outcome of this weekly regimen was not inferior to that of conventional regimens, and both adverse effects and compliance were significantly improved. Moreover, oropharyngeal cancer, extracapsular spread, and total radiation dose were independent prognostic factors for OS. The modified weekly regimen is a manageable protocol, and larger studies using this protocol should be evaluated.

## Figures and Tables

**Table 1:** Characteristics of patients.

Characteristic	Patients (*N* = 117)
Age (years)	
<65	101 (86.3)
≥65	16 (13.7)
Gender	
Male	112 (95.7)
Female	5 (4.3)
Primary site	
Oral cavity	47 (40.2)
Oropharynx	19 (16.2)
Hypopharynx	41 (35.0)
Larynx	7 (6.0)
Others^*^	3 (2.6)
Differentiation of tumor	
Well differentiated	61 (52.1)
Moderately differentiated	48 (41.0)
Poorly differentiated	8 (6.8)
Pathologic staging	
I	4 (3.4)
II	8 (6.8)
III	14 (12.0)
IV(a)	83 (70.9)
IV(b)	8 (6.8)
p16^INK4A^	
Positive	8 (44.4)
Negative	10 (55.6)
Detectable^†^	18
Extracapsular spread	
Positive	41 (35.0)
Negative	76 (65.0)
Regional lymph node involvement	
<2	54 (46.2)
≥2	63 (53.8)
Microscopic resection margin status	
Positive	53 (45.3)
Negative	62 (53.0)
Unknown^‡^	2 (1.7)
Tumor emboli	
Positive	55 (47.0)
Negative	60 (51.3)
Unknown^‡^	2 (1.7)
Perineural invasion	
Positive	64 (54.7)
Negative	51 (43.6)
Unknown^‡^	2 (1.7)
Lymphovascular invasion	
Positive	87 (74.4)
Negative	28 (23.9)
Unknown^‡^	2 (1.7)

^*^Others included two patients with carcinoma of unknown primary and one patient with nasal antrum cancer.

^†^In our institution, p16^ INK4A^ immunohistochemistry was checked since June 2012.

^‡^These were the cases of two patients with carcinoma of unknown primary.

**Table 2:** Compliance of patients.

Characteristic	Patients (*N* = 117)
Total radiation dose (Gy)	
≥60	111 (94.9)
<60	6 (5.1)
Number of weekly chemotherapy (cycle)	
≤4	14 (12.0)
5	15 (12.8)
6	48 (41.0)
7	37 (31.6)
8	3 (2.6)
Admission during CCRT	
Never	97 (82.9)
Admission more than 5 days	20 (17.1)

**Table 3:** Adverse events.

	Grade 1	Grade 2	Grade 3	Grade 4
Hematologic event				
Neutropenia^#^	57 (48.7)^#^	3 (2.6)^#^	0 (0.0)^#^	0 (0.0)^#^
Febrile neutropenia	0 (0.0)	0 (0.0)	1 (0.9)	0 (0.0)
Anemia	37 (31.6)	14 (12.0)	0 (0.0)	0 (0.0)
Thrombocytopenia	3 (2.6)	3 (2.6)	2 (1.7)	0 (0.0)
Nonhematologic event				
Nausea/vomiting	3 (2.6)	0 (0.0)	1 (0.9)	0 (0.0)
Skin	52 (44.4)	17 (14.5)	2 (1.7)	0 (0.0)
Mucositis	9 (7.7)	62 (53.0)	30 (25.6)	3 (2.6)
Xerostomia	3 (2.6)	0 (0.0)	0 (0.0)	0 (0.0)
Anorexia	0 (0.0)	29 (24.8)	6 (5.1)	0 (0.0)
Hyperbilirubinemia^∗#^	0 (0.0)	0 (0.0)	0 (0.0)	0 (0.0)
Acute renal injury	6 (5.1)	1 (0.9)	0 (0.0)	0 (0.0)
Neuropathy^#^	0 (0.0)^#^	0 (0.0)^#^	0 (0.0)^#^	0 (0.0)^#^

^*^The definition of hyperbilirubinemia is according to CTCAE v3.0. Grade 1: >ULN-1.5 × ULN; grade 2: >1.5–3.0 × ULN; grade 3: >3.0–10.0 × ULN; and grade 4: >10.0 × ULN.

**Table 4:** Univariate and multivariate Cox regression analysis for the prognostic factors of overall survival.

	Univariate		Multivariate	
	*P* value	HR (95% CI)	*P* value	HR (95% CI)
Age (years) ≥65	0.269			
Male	0.761			
Oropharynx	0.014	0.391 (0.185–0.829)	0.000	0.261 (0.116–0.586)
Extracapsular spread	0.012	2.443 (1.219–4.895)	0.007	2.709 (1.312–5.592)
Pathologic staging				
pT > 2	0.894			
pN > 1	0.472			
p16^INK4A^	0.583			
Regional lymph node involvement	0.501			
Resection margin status	0.891			
Tumor emboli	0.476			
Perineural invasion	0.611			
Lymphovascular invasion	0.892			
Total radiation dose ≥60 Gy	0.012	0.285 (0.107–0.761)	0.007	0.241 (0.086–0.679)
Weekly chemotherapy ≥7 cycle	0.152			

HR: hazard ratio; CI: confidence interval.

**Table 5:** Main trials on adjuvant treatments comparing chemoradiotherapy with radiotherapy alone after primary surgery.

Author (year)	Patients	Compared arm	Median follow-up (months)	Outcome
Bachaud et al. 1996^#^ [10]	83^#^	CP + RT vs. RT alone^#^	60.0^#^	2 y DFS rate, 68% vs. 44% (*P* < 0.02)^#^
				2 y OS rate, 72% vs. 46% (*P* < 0.01)^#^

Salama et al. 2007 [11]	114	Mito + Bleo + RT vs. RT alone	32.2	2 y DFS rate, 76% vs. 60% (*P* = 0.099)
				2 y OS rate, 74% vs. 64% (*P* = 0.036)

Cooper et al. 2004 [1]	459	CP + RT vs. RT alone	45.9	2 y DFS rate, 54% v. 45% (*P* = 0.04)
				2 y OS rate, 64% vs. 57% (*P* = 0.09)

Bernier et al. 2004 [2]	334	CP + RT vs. RT alone	60.0	5 y PFS rate, 47% vs. 36% (*P* = 0.04)
				5 y OS rate, 53% vs. 40% (*P* = 0.02)

Current study	126	CP + uracil-Tegafur + RT	30.0	2 y DFS rate, 70.9%
				2 y OS rate, 79.5%

Mito: mitomycin C; Bleo: bleomycin; RT: radiotherapy; CP: cisplatin; LRC: locoregional control; DFS: disease-free survival; OS: overall survival.
